# The role of temperature-induced effects generated by plasmonic nanostructures on particle delivery and manipulation: a review

**DOI:** 10.1515/nanoph-2022-0014

**Published:** 2022-04-05

**Authors:** Domna G. Kotsifaki, Síle Nic Chormaic

**Affiliations:** Light-Matter Interactions for Quantum Technologies Unit, Okinawa Institute of Science and Technology Graduate University, Onna-San, Okinawa, Japan; Natural and Applied Sciences, Duke Kunshan University, 8 Duke Ave, Kunshan, Jiangsu, China

**Keywords:** biomolecules, convection effects, nanoparticles, plasmonic nanostructures, thermophoresis

## Abstract

Plasmonic optical tweezers that stem from the need to trap and manipulate ever smaller particles using non-invasive optical forces, have made significant contributions to precise particle motion control at the nanoscale. In addition to the optical forces, other effects have been explored for particle manipulation. For instance, the plasmonic heat delivery mechanism generates micro- and nanoscale optothermal hydrodynamic effects, such as natural fluid convection, Marangoni fluid convection and thermophoretic effects that influence the motion of a wide range of particles from dielectric to biomolecules. In this review, a discussion of optothermal effects generated by heated plasmonic nanostructures is presented with a specific focus on applications to optical trapping and particle manipulation. It provides a discussion on the existing challenges of optothermal mechanisms generated by plasmonic optical tweezers and comments on their future opportunities in life sciences.

## 1 Introduction

Decades after their first demonstration [1, 2], conventional optical tweezers are valuable tools for isolating and manipulating small particles [3–14]. Their versatility has been proven by several applications in the microscale regime. However, restrictions arise due to the diffraction limit that reduces their applicability in the nanoscale. To enable the trapping and manipulation of nanosized particles, various plasmonic trapping schemes [15–23] have been demonstrated. When a strong confinement of incident light along the metal surface occur (resonant conditions), plasmonic nanostructures create highly localised and enhanced electromagnetic fields that produce narrow and deep trapping potential wells. These characteristics facilitate trapping nanoscale particles with high stability [24–32].

In addition, upon illumination, the plasmonic nanostructures exhibit Joule heating owing to strong absorption of the light in metals [33, 34]. The heating becomes significant on resonance, leading to an abrupt temperature gradient at the interface between the nanostructure and the surrounding medium [33]. This local temperature increase generated by the heated nanostructures can induce several effects, such as thermal convection and thermophoresis, that can influence the optical trapping process.

Thermal convection is a type of fluidic flow caused by buoyancy due to the density gradient of the fluid. Thermophoresis is the migration of suspended particles along the gradient of a local non-uniform temperature profile [35]. Such particle motion has been observed in colloidal suspension [36–38], nanoparticles [39–42], polymers [43, 44], micellar solutions [41, 45], DNA [46] and proteins [47]. The possible relevance of thermophoresis to the origin of life is also interesting [48–50]. In this case, the replication of information-bearing molecules, such as DNA and RNA, and the selection under thermodynamic equilibrium on early earth may allow us to understand molecular assembly and evolution [48–50].

Optothermal effects generated by heated metallic nanostructures have played key roles in recent studies [51–55]. In the literature, several review papers [56–60] have addressed the role of thermal effects on mass transportation and manipulation. In this review, the physical mechanism of temperature gradient-induced effects is highlighted briefly in order to elucidate their contribution in optical trapping applications and in particle manipulation processes. Here, when we refer to optothermal effects, we mean the phenomena that arise from the combination of optics and temperature gradients that lead to mass transportation. We comprehensively review relevant fluid mechanics behaviours such as Rayleigh–Bénard fluid convection [61], the Marangoni–Bénard fluid effect [62, 63] and thermophoresis [58, 60]. We further discuss some examples of applications of these effects in the life sciences. Finally, an overview of possible future research directions of optothermal effects is provided.

## 2 Plasmon-assisted thermally generated effects

### 2.1 Heat generation in plasmonic nanostructures

When light strikes a metallic nanostructure, the amplified movement of the conduction electrons within the metal increases the frequency of collisions with the lattice atoms, leading to Joule heating. This process results in a heat source density that is defined as [64]:

(1) $$q\left(r,t\right)=\frac{1}{2}R\left(J^{⋆}\left(r,t\right)\cdot E\left(r,t\right)\right),$$

where **J**(**r**,*t*) is the induced current density (charge per unit time and unit area) that generates the energy dissipation via the Joule effect, **E**(**r**,*t*) is the electric field inside the metallic nanostructure, R and ^⋆^ represent the real part and the complex conjugate, respectively. The current density can be expressed in terms of the polarisation vector **P**(**r**,t) [64]:

(2) $$J\left(r,t\right)=-i\omega P\left(r,t\right)=-i\omega \varepsilon_{o}\varepsilon \left(\omega \right)E\left(r,t\right),$$

where *ɛ*_o_ is the dielectric permittivity in vacuum, *ɛ*(*ω*) is the relative permittivity of the nanostructures and *ω* is the optical frequency. The heat source density can also be defined by [64]:

(3) $$q\left(r,t\right)=\frac{\omega }{2}\varepsilon_{o}I\left(\varepsilon \left(\omega \right)\right)|E\left(r,t\right){|}^{2}.$$

where I is the imaginary part. The heat source density within the nanostructure is proportional to the square of the amplitude of the electric field and the total heat power is defined by [64]:

(4) $$Q\left(r,t\right)=\frac{\omega }{2}\varepsilon_{o}I\left(\varepsilon \left(\omega \right)\right)\underset{V}{\int }|E\left(r,t\right){|}^{2}dr,$$

where the integral covers the entire nanostructure volume, *V*. It should be noted that, for small nanostructures with dimensions less than the mean free path of the heat carrier, the heat transportation deviates from that predicted by Fourier heat conduction theory [65]. The Fourier law is valid when there are enough scattering events around the nanostructure such that the heat carriers excited by the heat-generating nanostructure can exchange energy with the surrounding medium [65]. With decreasing nanostructure size, very few scattering events occur around the heated nanostructure. In this region, the temperature is high inside the nanostructure and the heat carrier transportation is non-local. Therefore, a non-equal temperature at the interface between nanostructures and the surrounding medium can be generated. Such a temperature non-equality or jump was predicted for heat conduction across a thin film [66]. This heat transportation process between the nanostructure and the surrounding medium may create a large heat flux in the fluidic system.

**Figure 1:**
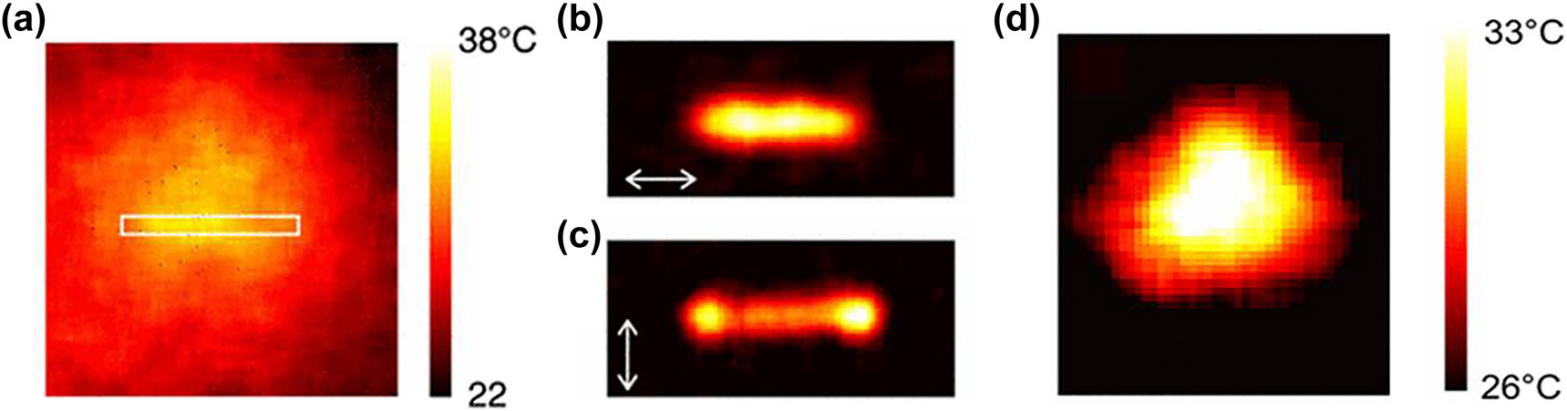
Temperature profile around plasmonic nanostructures. (a) Temperature distribution around a gold nanowire. Heat source density for (b) parallel and (c) perpendicular polarisations, respectively, (d) heat source density associated with an array of nanoholes. Figures reproduced with permission from [33], Copyright 2010, APS Physics.

Extensive studies on the heat source density of plasmonic nanostructures revealed that the temperature gradient depends on the nanostructure morphology and can be non-uniform in contrast with the temperature distribution, which is uniform owing to the fast thermal diffusion in metals [33, 64, 67, 68]. The temperature distribution around a gold nanowire was measured using a thermal microscopy technique as shown Figure 1(a). The authors noted that, although the temperature diffuses from the gold nanowire, the heat origin is restricted to the metal for both polarisations considered (Figure 1(b) and (c)) [33]. They also mentioned that a way to increase the heat generation in metals is by patterning the metallic structure [33]. Figure 1(d) shows the heat generated around a structure patterned with an array of nanoholes [33]. Note that typical methods widely used for nanoscale temperature measurements utilise temperature-dependent fluorescent dyes, such as Rhodamine B [69] or Alexa Fluor dyes [70], with the latter preferred because it avoids the metal photoluminescence found in gold layers below 600 nm [64].

### 2.2 Rayleigh–Bénard fluid convection

**Figure 2:**
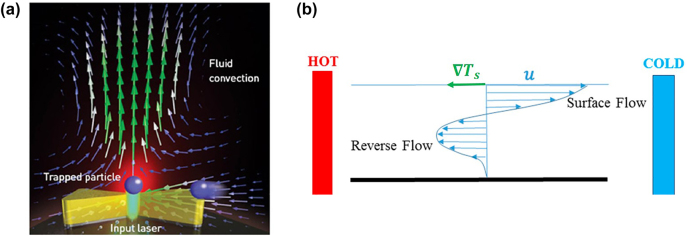
Convection effects. (a) Schematic illustration of natural convection flow. The illuminated plasmonic nanostructure heats the fluid and induces a local temperature gradient. Green arrows represent the fluid velocity. Figure reproduced with permission from [78], Copyright 2015 OSA. (b) Schematic of Marangoni flow section driven by a surface temperature gradient, ∇*T*_*s*_. The tangential surface velocity vector of the Marangoni flow, *u*, can also be observed. Figure reproduced with permission from [79], Copyright 2016 MDPI.

The temperature generated inside the nanostructure can be described by the heat diffusion equation [58, 64]:

(5) $$\rho c_{p}\frac{\partial }{\partial t}T\left(r,t\right)-\kappa \nabla^{2}T\left(r,t\right)=q\left(r,t\right),$$

where *ρ* is the metal density, *c*_p_ the specific heat capacity at constant pressure of the metal and *κ* the thermal conductivity of the metal. The temperature distribution in the surrounding medium can be defined as [58, 64]:

(6) $$\begin{array}{ll} & \rho_{1}c_{1p}\left[\frac{\partial }{\partial t}T\left(r,t\right)+\nabla \left(T(r,t)v\left(r,t\right)\right)\right]\\ & \;-\kappa_{1}\nabla^{2}T\left(r,t\right)=0, \end{array}$$

where *ρ*_1_, *c*_1p_ and *κ*_1_ are the density, specific heat capacity at constant pressure and thermal conductivity of the medium, respectively. The second term in the bracket on the left-hand side is the nonlinear convection term that depends on the velocity of the fluid, and **v**(**r**,t) is the surrounding fluid velocity. Therefore, as the temperature of the nanostructure increases, the surrounding fluid density decreases, leading to heat generation via an upward convection flow (Archimedes force) known as toroidal-shaped flow, as shown in Figure 2(a). The velocity field profile can be determined by solving the incompressible Navier–Stokes equation [71] as follows:

(7) $$\rho_{s}\left(u\left(r\right)\cdot \nabla \right)u\left(r\right)+\nabla p\left(r-\eta \nabla^{2}u\left(r\right)\right)=F,$$

where ∇⋅**u** = 0, **u**(**r**) represents the surrounding fluid velocity and **F** is the force per unit volume acting on the fluid element. For nonisothermal flow problems [52, 61, 72], the Boussinesq approximation is implemented, and the buoyancy-driven force term is defined as [72]:

(8) $$F=g\beta \left(T\right)\rho_{s}\left[T\left(r,t\right))-T_{\infty }\right]z,$$

where *g*, *β* and *T*_∞_ are the gravitational constant, the thermal expansion coefficient of water and the initial temperature, respectively. The natural or Rayleigh–Bénard convection originates from fluid gravity induced by the temperature-dependent density of the fluid [73]. The time taken for Rayleigh–Bénard fluid convection to reach a steady state depends on the size and morphology of the nanostructure [67, 74], and can be shortened by reducing the solution chamber height, where particles was placed in and the optical trapping occurred, in the *z*-direction to less than 50 μm [61, 75]. A single light-absorbing nanostructure can provide weak natural convection that sometimes is not sufficient for the delivery or manipulation of nanoscale particles [61]. For instance, for a double nanohole the maximum predicted fluid velocity exceeds 7 nm/s [76], even if the temperature reaches boiling point [61]. In other words, the particle needs a long time to diffuse into close proximity of the nanostructure before it can be trapped. Natural convection, which assists particle delivery and manipulation, can be increased by utilising an array of nanostructures that increases the natural convection fluid velocity [32, 77].

### 2.3 Bénard–Marangoni fluid convection

Marangoni or thermocapillary convection is a convection flow that can occur at liquid–gas interfaces under high temperature inputs [63, 80, 81]. It is caused by the temperature-dependent surface tension at the liquid–air interface, where the fluid molecules are dragged towards regions with higher surface tension. In general, temperature gradients perpendicular to the liquid surface can create fluid circulation inside the liquid, while temperature gradients tangential to the liquid surface produce a surface tension gradient along the surface and induce surface flow from low to high surface tension regions [79]. In most cases, the liquid surface tension decreases linearly with increasing temperature for small temperature perturbations. Therefore, if a spatial temperature profile is imposed on a liquid surface, a shear stress, ***τ***_*s*_, is generated, oriented from the hot to the cold side, and is proportional to the temperature gradient [62]. For a Newtonian fluid, the shear stress is defined as [62]:

(9) $$\tau_{s}=\mu \frac{\partial u_{s}}{\partial N}=-\sigma_{T}\nabla T,$$

where *μ* is the dynamic viscosity of the fluid, **N** is the surface normal vector, ***σ***_*T*_ is the temperature coefficient of the surface tension which can be calculated according to the Eötvö law [82]. Figure 2(b) shows a schematic cross section of Marangoni flow driven by a surface temperature gradient, where the shear stress (blue lines) and flow vector can be observed and are in directions opposite to the temperature gradient (∇*T*_*s*_) [62]. The figure also shows that the tangential surface velocity, $\vec{u}$ , is directed towards the cold region near the liquid–air interface and then flows back to the hot side via subsurface flow (Figure 2(b)). Note that the depth of this movement depends on the length and height of the flow in the chamber. A reverse Marangoni effect can be observed, for example, in dilute alcohol/water mixtures, where the temperature gradient on the gas–liquid interface drives the surrounding liquid from cold to hot regions. Namura et al. [63] reported that the solute component influences the convection and its concentration gradient along the liquid–air interface modifies the surface tension similar to the temperature gradient. Because of its large working range and high velocity, Marangoni convection is applicable to both nanoscale and macroscale particles.

## 3 Thermophoretic force

The mass diffusion flow occurring along a temperature gradient has been known for a long time [35]. The directional mass flow of target objects, such as particles and biomolecules, under an external temperature gradient can be described by [83]:

(10) $$J=-cD_{T}\nabla T-D\nabla c,$$

where *c* is the mass concentration, *D*_*T*_ is the thermophoretic mobility that describes the coupling between heat and particle flow [83, 84], and *D* is the Brownian diffusion coefficient. The first term on the right-hand side of Eq. (10) denotes the migration of the mass driven by the temperature gradient and the second gterm refers to the flow driven by the concentration gradient. Under steady-state conditions, where the total maximum flux is zero (**J** = 0), the concentration gradient of the suspended mass is defined as [83]:

(11) $$\nabla c=-cS_{T}\nabla T,$$

where the Soret coefficient, *S*_*T*_, is the ratio of the thermal and Brownian diffusion coefficients (*S*_*T*_ = *D*_*T*_/*D*) and is the key point for thermophoresis. The magnitude of the Soret coefficient can indicate the particle concentration gradient in a temperature field. Depending on the sign of the Soret coefficient, the suspended particles within the temperature gradient can move towards the cold region (when the sign is positive: *S*_*T*_ > 0) and are labelled as thermophobic or can move towards the hot region (when the sign is negative: *S*_*T*_ < 0) and are labelled as thermophilic. In Table 1, the Soret coefficients for several particle sizes under various conditions are summarised.

**Table 1:** Soret coefficients of several particles.

Particle type	Radius of particle	Surrounding medium	Thickness of chamber (μm)	Temperature (°C)	Soret coefficient (K^−1^)
Polystyrene (PS)	2.5 μm	Water	7.70	27	163.9 [85]
Polystyrene (PS)	215 nm	Water/1 mM Na*OH*_*x*_*Cl*_1−*x*_ (*x* ∼ 0.1)	100	41	∼1.90 [86]
Dye-doped PS	50 nm	Water/10 mM Tris/2% PEG			∼−0.20 [87]
Carboxylated melamine	1.35 μm	Water	7.70	27	−30.4 [85]
Carboxylated dye-doped PS	250 nm	Water/pH = 7.6/Tris	10		4.60 [88]
Carboxylated PS	100 nm	Water/1 mM Tris	10/20		0.70 [36]
Carboxylated PS	13 nm	Water	300	20	∼−0.38 [89]
Carboxylated PS	13 nm	Water	300	70	∼0.02 [89]
Carboxylated PS	11 nm	Water/pH = 7.8/1 mM–Tris HCl	20	25	∼−0.13 [84]
Silica Ludox	12.3 nm	Water/pH = 7/0.1% NaCl		25	∼−0.21 [38]
PbS	8 nm	Cyclohexane	1000		−4.0 [90]
α-Synuclein fibril	100 nm	Water/pH = 7.4/1 mM Tris	50	∼27	∼0.14 [91]
α-Synuclein oligomer	7.5 nm	Water/pH = 7.4/1 mM Tris	50	∼29	∼0.08 [91]
α-Synuclein monomer	2.8 nm	Water/pH = 7.4/1 mM Tris	50	∼29	∼0.03 [91]
Lysozyme	7 kDa	Water/pH = 4.55/100 mM NaCl		27	0.0013 [37, 92]
SYBR *DNA*	5.6 kbp	Water/pH = 7.8/10 mM Tris	25/500	24	0.14 [46]
SYBR green I *λ*-DNA	48.5 kbp	Water/pH = 7.8/10 mM Tris	10		0.40 [93]

It should be noted that the sign of the Soret coefficient depends on a large number of factors such as the particle size [36, 38, 40, 47, 84, 89], surrounding temperature [47], Debye length [36, 94], solvent properties [40, 95], particle surface charge [96], and the particle hydrophilicity [96, 97]. For example, silica [98] and polystyrene particles [84] of similar size and surface charge diluted in an ionic solution can display Soret coefficients of opposite signs. In the case of polymers with molecular weights in the scale of approximately 10^5^ (g/mol), the Soret coefficient is in the range of 0.02–0.5 K^−1^ [83, 84]. The dependence of thermal diffusion, *D*_*T*_, and the Soret coefficient on the physiochemical properties of solvents has also been studied extensively [99, 100]. Hartung et al. [100] measured the thermal diffusion of polystyrene in different solvents and concluded that it was proportional to the solvent viscosity. For charged particles in an electrolyte solution, the thermoelectric effect provides a nonlocal driving force that can influence their motion [96]. This particle motion is mainly determined by the sign of the particle surface charge [96]. The interactions between particles and solvents can be altered by the thermal expansion of the solvent under temperature gradients that lead to another contribution to the thermophoresis. For example, hydrophilic particles prefer to remain in the cold region where the water density is higher while hydrophobic particles remain in the hot region where the water density is lower [97]. In the following, the underlying physics of thermophoresis is described briefly and various effects on colloidal particles are discussed.

### 3.1 Dispersion force

A particle suspended in a non-uniform liquid interacts with it through van der Waals forces [83]. Under the influence of a temperature gradient, dispersion forces can be generated from the density gradient of the liquid [83] and result in a motion of the suspended particles from hot to cold regions [83]. These forces decay quickly with distance and are strongest close to the particle surface [83]. Generally, common liquids expand upon heating, with the exception of water below 4 °C because it creates an antiparallel density gradient along the temperature gradient [83, 104, 105]. Therefore, the colder liquid region with a higher particle density experiences a stronger van der Waals attraction from the particles than the hot side. This leads to a slip flow, which drives the particles towards the cold region.

The thermophoretic drift velocity due to the dispersion force is defined as [83]:

(12) $$u=-\frac{2\beta H}{9\pi \eta d_{o}}\nabla T,$$

where *H* is the Hamaker constant of liquid molecule–particle interactions, *η* is the liquid viscosity and *d*_o_ is the molecular length scale of the liquid. It has been reported that the magnitude of the drift velocity can be several micrometres per second [83, 101]. From Eq. (12), the dispersion forces become significant in liquids with a high expansivity, *β* and low viscosity, *η*. In an isotropic liquid, the dispersion forces on both sides of a particle cancel each other and the drift velocity arises from a spatial variation of the liquid concentration [83]. For charged particles in an aqueous solution, in the presence of a temperature gradient, the contribution of dispersion forces to particle motion is minor compared to entropy-driven forces [83, 101].

### 3.2 Entropy-driven effect

**Figure 3:**
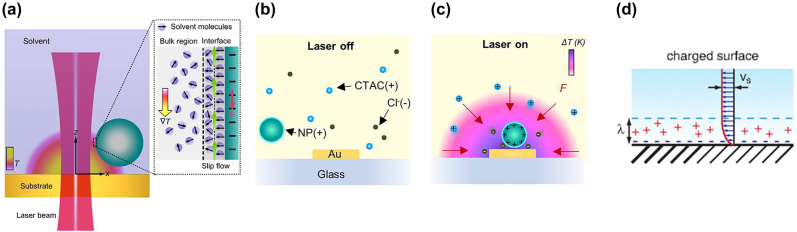
Optothermophoretic effects. (a) Schematic of thermophoretic particle trapping at the hotspot of a gold substrate upon illumination by a laser beam. Inset: A layered structure of solvent at the particle–solvent interface where the interfacial-entropy-driven force, *F*_i_ has been generated by the induced slip flow (green arrows) under a temperature gradient field ∇*T*. Figure reproduced with permission from [101], Copyright 2018 ACS publications. (b) Dispersion of positively charged nanoparticles and cetyltrimethylammonium chloride (CTAC), ions surrounding the gold nanostructure when the laser beam is off, and (c) thermophoresis-induced redistribution of ions in the liquid environment when the laser beam is on. Upon illumination, the generated temperature gradient creates a thermo-electric force, *F*, which transports and traps the nanoparticles at the centre of the nanostructure. Figures reproduced with permission from [102], Copyright 2018 ACS publications. (d) Schematic illustration of the thermo-osmotic velocity profile close to a charged solid boundary with excess enthalpy within an interaction length, *λ*, which is the length scale of the depletion interaction among colloids at equilibrium. Figure reproduced with permission from [103], Copyright 2016 APS Physics.

In addition to the van der Waals forces, the molecular structure of the liquid influences the motion of the suspended particles. As described in the model proposed by Bockris et al. [106], water molecules can be absorbed onto the charged surface of the particles to form a dipole layer owing to electrostatic interactions at their interface [107] (Figure 3(a)). Specifically, in the first layer, the orientation of the water molecules is aligned with the electric field from the charged surface. In the second layer, they are partially oriented towards the particle surface, while in the third layer and beyond they become disordered (inset Figure 3(a)). If we apply a temperature gradient, the orientation of the interfacial water molecular dipoles at the hot side of the particle becomes disordered owing to the increased interfacial entropy. This thermal perturbation leads to the generation of a large permittivity gradient parallel to the temperature gradient [101, 106]. According to Anderson’s theory, the thermal diffusion coefficient/thermophoretic mobility, *D*_*T*_, is proportional to the permittivity gradient term, ∂In*ɛ*/∂In*T*, which for pure water is −1.4 C^2^ N^−1^ K^−1^ m^−2^ [89]. This suggests a positive thermophoretic mobility, *D*_*T*_ [89, 108]. However, at the particle–water interface, the permittivity gradient term becomes positive and the sign of *D*_*T*_ is reversed, driving the particle migration from cold to hot. In general, the electric double layer formed at the particle–liquid interface can be determined by zeta potential and surface tension measurements [58]. The working mechanism is described in Refs. [60, 109].

### 3.3 Thermo-electric force

Beyond pure liquids, particles can be suspended in more complex liquids with ions or solute molecules, see Figure 3(b) and (c). Thermal forces are very sensitive to electrolyte composition owing to the Seebeck effect [95, 109], [110], [111]. For example, in sodium hydroxide (NaOH) solutions, the anion groups, OH^−^, tend to accumulate at the cold side because the heat transport for anions is much higher than for cations, Na^+^ [86, 109, 112]. This results in the spatial separation of positive and negative ions [112], moving the suspended particles to the hot side (Figure 3(c)). In this case, thermophoresis shows a similar charge effect behaviour to the Hofmeister series of protein interactions [86, 113]. Thermo-electric forces can be generated by the polarisation of water molecules [114, 115]. Relevant studies have shown that water polarisation and charge separation play key roles in the formation of thermo-electric fields in different salt solutions under temperature gradients [114–116]. Cetyltrimethylammonium chloride (CTAC) is a cationic surfactant used to manipulate metallic nanoparticles, can also significantly influence the thermophoretic mobility of the particle due to its surface charge modification [117, 118].

### 3.4 Thermo-osmotic effect

Thermo-osmosis is a phenomenon that occurs on interfaces of non-uniform temperature, see Figure 3(d). An interaction layer is generated at the interface between the nanostructure and the liquid, defined by specific interactions (*i.e.* electrostatic, hydrogen bonds, van der Waals etc.), of the surface with the liquid. These interactions can be characterised by an enthalpy in the interfacial layer compared to the bulk [103, 119]. In the case of a non-isothermal surface, the enthalpy varies across the interface resulting in a stress in the interfacial layer, leading to a thermo-osmotic surface flow. In the case where a particle is suspended in a liquid with a temperature gradient, this stress integrated over the particle’s surface does not vanish, and drives the particle in the opposite direction of the boundary flow [103, 119]. In a system containing particles suspended in liquid where ions are present, the electric double layer around particle surfaces is influenced by ionic transport owing to the temperature gradient [83]. Specifically, ions accumulate around the charged surface of the particle, creating hydrostatic pressure within the diffusion layer [83]. Under local heating, the ionic cloud around the charged surface is distorted. As a result, the charged solvent near the surface flows towards the hot region and the particle drifts along the direction opposite to that of the drift velocity, defined as [83]:

(13) $$u=-\frac{\varepsilon \zeta^{2}}{3\eta }\frac{\nabla T}{T},$$

where *ζ* is the zeta potential, which is proportional to the Debye length, and *ɛ* is the solvent permittivity. Therefore, the magnitude of the thermo-osmotic effect is significantly influenced by electrolyte salinity.

### 3.5 Depletion force

**Figure 4:**
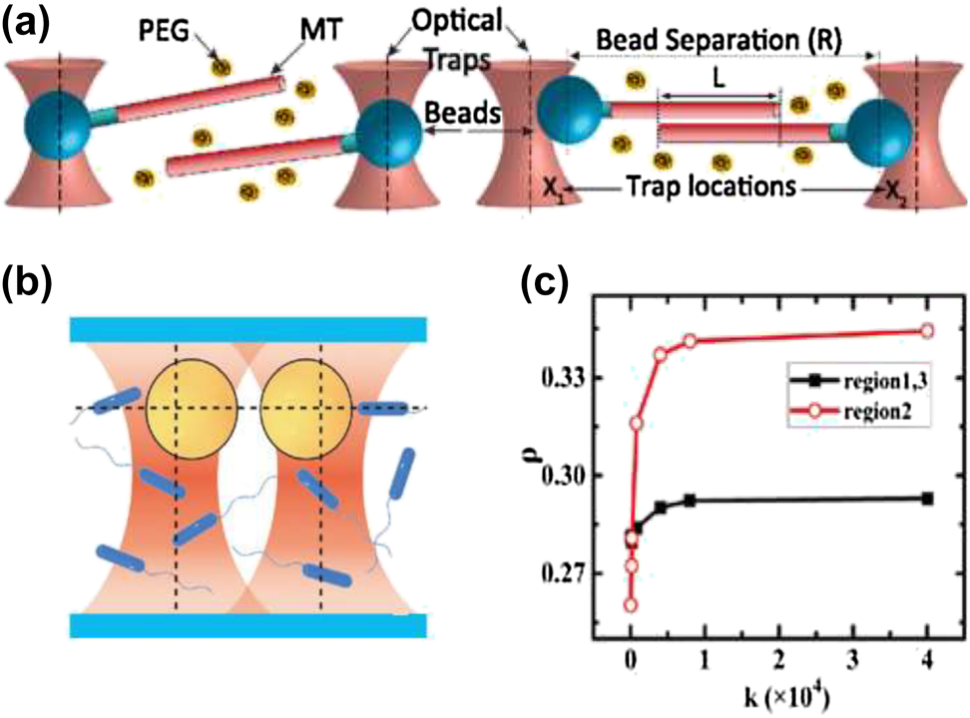
Depletion force. (a) Schematic illustration of optically trapped bead-microtubules (MT) filament complexes (left side). The MTs were suspended in non-absorpting polymers, *i.e.* polyethylene glycol (PEG). The right side shows the depletion force that creates attractive interactions, which pull the trapped beads close together, away from the trapping point. Figure reproduced with permission from [121], Copyright 2015 APS Physics. (b) A sketch of optically trapped spheres of 3 μm diameter in bacteria solution which induce attractive interactions by the depletion mechanism. (c) The theoretical force exerted on the two fixed spheres, *F*_eff_, versus the distance between them for various trap stiffnesses, *k*. Figures reproduced with permission from [122], Copyright 2020 APS Physics.

The solute molecule–particle interactions play a major role in the thermophoretic behaviour of suspended particles [87, 120], [121], [122]. Molecules in a solvent have a smaller size than the particles and a high Brownian diffusion coefficient; thus, under a temperature gradient, they can accumulate with large velocity in a cold region [57]. Consequently, the concentration gradient of molecules, ∇*c*, orients towards the opposite direction of the temperature gradient, resulting in an osmotic pressure that pushes the suspended particles from the cold to the hot region. This force is called depletion [49, 87, 88, 103, 120, 123] and can be adjusted by changing the concentration, for instance, in polyethylene glycol (PEG) solution. Such forces have been investigated extensively because of the precise control of particle trapping and assembly by tailoring the characteristics of the depletant molecules (Figures 3(d) and 4) [120, 124].

For example, a high concentration of 100 nm particles was demonstrated under a temperature gradient in a PEG solution [87]. The PEG molecules moved away from the laser beam to the cold region due to thermophoresis [87]. This movement generated a depletion force capable of driving the suspended particle to the laser hot spot [87]. The depletion interactions between a pair of microtubule cytoskeletal filaments occurred as the depleting polymer was smaller than the colloid (Figure 4(a)) [121]. Recently, the active depletion force on a particle of 3 μm diameter from the surrounding bacteria in steady state was measured, showing a way to control the thermophoretic properties of colloids (Figure 4(a) and(b)) [122]. In heat-mediated optical manipulation schemes, the concentration of the small molecules could be achieved by their thermal migration in the presence of the temperature gradient fields that are controlled by the nanostructure geometry [64]. Therefore, the plasmonic substrates could be used as heating sources, which generate a temperature gradient when illuminated. The particle drift velocity can be defined as [87]:

(14) $$u=\frac{k_{B}T}{3\eta }\left(S_{T}^{P}-\frac{1}{T}\right)\lambda^{2}c\nabla T,$$

where *λ* is the interaction distance between colloids in equilibrium as noted in Figure 3(d), and $S_{T}^{P}$ is the Soret coefficient of the particle.

## 4 Particle manipulation through effects generated by heated metallic nanostructures

Rayleigh–Bénard convection is a type of flow attributed to the density gradients of fluids and can be generated when absorbing substrates, colloids, or solvents are illuminated. It usually manipulates suspended particles through the Stokes drag force. In addition, it has been widely applied to nanoparticle transport towards plasmonic trapping hotspots due to its long working range [61] and the fact that it can overcome the diffusion limit in optical trapping systems [125]. However, optical manipulation precision of single nanoparticles by natural convection is low [61]. On the other hand, Marangoni convection arises from the surface tension gradient, which can be induced by bubble formation [126] or concentration gradients of chemicals [81]. Marangoni convection occurring from localized optothermal bubbles can be used for fast and large-scale particle manipulation in optical tweezers [125].

When laser heated in a solution, suspended particles are influenced by the induced temperature gradient and migrate towards either the hot or the cold region depending on the sign of the Soret coefficient. By controlling the heating, particles can be trapped via thermophoresis [127]. For instance, a ring-like temperature field was shown to trap a single amyloid fibril [128]. Particle trapping can also be achieved by entropy-driven forces [129], where the trapping stability can be improved by engineering the particle hydrophilicity, particle surface charge, ionic strength on interfacial structures or solvent type [101]. For an electrolyte solvent, the thermophoretic migration of cations and anions leads to an ionic spatial redistribution to establish thermoelectric fields [95]. These fields can be used to optically manipulate a variety of particle materials, sizes and shapes [95, 109]. In addition, exploiting the depletion forces between particle–particle or particle–liquid interfaces, particle assembly and printing have been demonstrated [129]. Depletion forces are suitable to generate local concentration gradients of small molecules around suspended particles. They are applicable for various particles, such as dielectric particles [87], biomolecules [88], etc. and are highly dependent on the size as well as concentration of depletants [88].

Each of the above mentioned effects has advantages and limits in optical manipulation depending on the applications. For example, natural convection has low position accuracy, but is can be used in conjunction with thermophoresis [46], the latter enabling stable and dynamic particle manipulation [129, 130]. Depletion forces tend to have a relatively large working range [87] while thermo-electric forces have low operational power [57]. Therefore, it is challenging to compare them in a comprehensive way. Thence, precise control of the optical forces, thermal effects and thermophoresis can enable remote, noncontact and versatile manipulation of several particles using low a trapping laser power. In the following, we present some related applications.

### 4.1 Influence on photothermal convection by using several substrates

Gold, which is widely used to fabricate plasmonic nanostructures, requires an adhesion layer to adhere firmly to a transparent substrate. It has been shown that the absorption losses from the adhesion layer can dampen the enhancement factor of single-molecule fluorescence [131, 132], increase the local temperature [54] and affect the nanotrapping potential well [133]. Jiang et al. [54], measured the local temperature inside a 300 nm diameter nanohole milled in 100 nm gold film. The authors studied the influence of several adhesion materials to explore the heat generation by varying the material of the adhesion layer and its thickness.

**Figure 5:**
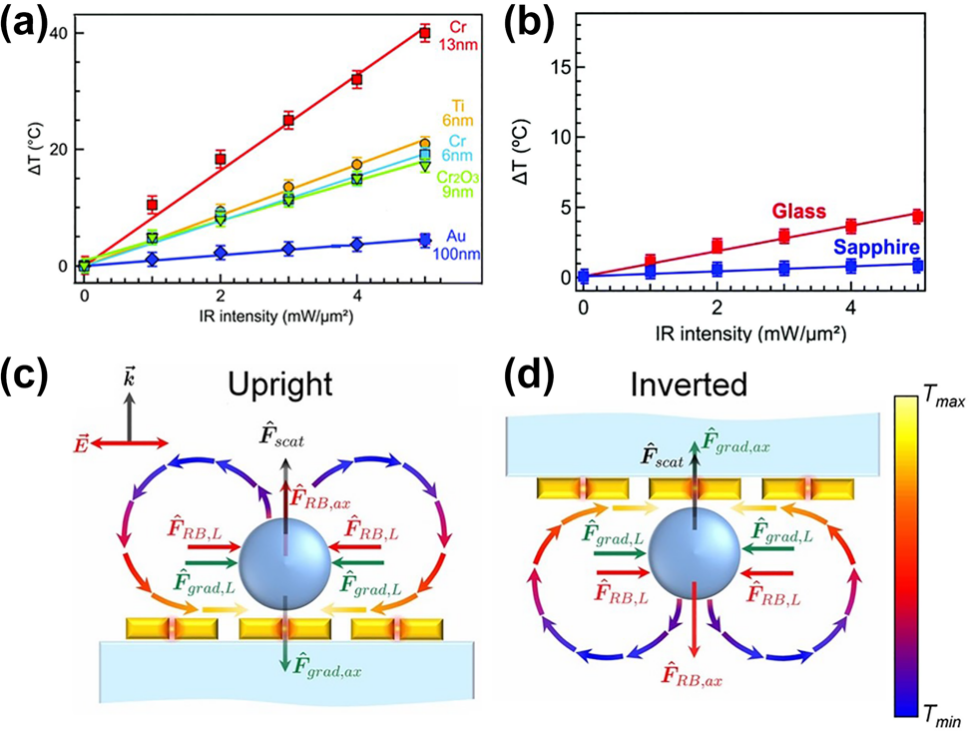
Plasmonic heating of various substrates. (a) Temperature increase in a 300 nm diameter single nanohole plotted against laser intensity and as a function of adhesion layer material and its thickness. (b) Temperature increase in a glass (red) and sapphire (blue) substrate versus laser intensity. Figures reproduced with permission from [54], Copyright 2020. The Royal Society of Chemistry. A schematic of (c) upright and (d) inverted configurations, where *F*_scat_ is the optical scattering force, *F*_grad_ and *F*_RB_ are the gradient and the thermal forces, respectively, and *L* and *ax* indicate laterally and axially directed forces, respectively. The arrows indicate the direction of natural convection with colour representing the relative temperature, *T*. Figures reproduced with permission from [133], Copyright 2012 The Optical Society.

Figure 5(a) shows that a 13 nm chromium (Cr) adhesion layer provides the maximum temperature increase, leading to the highest temperature magnitude of 40 °C at 5 mW/μm^2^ incident light intensity. The authors observed similar temperature behaviour [54] by using equal thickness adhesion layers for titanium (Ti) and chromium [54]. Note that in case of a chromium oxide (Cr_2_O_3_) layer a low temperature increase owing to low absorption can be provided compared to a chromium layer. An effective way to control the plasmonic-thermal effects was to use a thin adhesion layer of Cr_2_O_3_ with a sapphire substrate [54] (Figure 5(a) and (b)). Figure 5(b) shows that the heat dissipation in the sapphire substrate reduces the temperature increase compared to that in a glass substrate by four times [54].

Roxworthy et al. noted that the titanium adhesion layer provides up to 30% greater trap stiffness and trapping efficiency compared to a chromium layer of equal thickness [133]. They also investigated the modification in the trapping performance due to the nanostructure orientation [133]. They mentioned that the upright configuration (Figure 5(c)) results in a quasi-three-dimensional optical trap and the particle should be within 20 nm of the plasmonic nanostructure surface in order to be trapped, owing to Rayleigh–Bénard convection. In the inverted configuration (Figure 5(d)) a two-dimensional optical trap can be produced that enables efficient trapping of particles with diameters less than 300 nm. Two years later, the same research group used a 9 × 9 array of gold bowtie nanostructures on an optically absorptive indium-tin-oxide (ITO) substrate to theoretically and experimentally study the convection flow velocity [52] under on and off resonance conditions and to determine its variation by changing the ITO thickness. The authors showed that, on resonance the fluid velocity of the nanostructure was 10 nm/s using bare SiO_2_, while it increased by one order of magnitude when using an ITO adhesion layer. By varying the ITO thickness, they achieved the highest possible convection velocity for a nano-antenna system [52]. Furthermore, the authors also distinguished between the influence of the natural and thermoplasmonic convection on the trapping process [52]. Natural convection, stems from an instability in a microwell with a uniformly heated bottom surface that requires a large temperature across the whole fluid microwell to induce the instability as well as a Rayleigh number exceeding a certain threshold [134]. The latter case can be generated by spatially non-uniform heating of metallic nanostructures and only requires a Rayleigh number larger than zero. Recently, Yang et al. [135] demonstrated an alternative approach to suppress photothermal convection by using silicon carbide as a substrate. The authors noted that the gold-coated silicon carbide substrate can reduce the convection flow by a factor of seven compared to that obtained by the gold-coated glass substrate [135].

### 4.2 Rayleigh–Bénard fluid convection assists the trapping process

Natural convection flow can be applied to manipulate particles with various sizes particles [52, 61, 134, 136]. For example, Roxworthy et al. used the thermoplasmonic convection generated by an array of gold bowtie nanostructures to optically trap and sort differently sized polystyrene particles [137]. The authors observed that, at low laser power, a single particle was trapped under the influence of the gradient force near the bowtie nanostructure [137]. As the trapping laser power increased, convection flow pushed the particles away from the laser focus spot and trapped them as a cluster [137]. Single cancer cell analysis was demonstrated by combining the optical forces and thermoplasmonic convection that was achieved by illuminating a gold-coated microwell array [138]. Each microwell operated as a heat chamber with constant temperature for biological reactions, such as isothermal recombinase polymerase amplifications [138]. Natural convection can also be applied to achieve 3D pitch rotation of single microparticles and cells [139]. The generation of pitch-rotation motion in an optical tweezers system has remained elusive due to the complexities of generating high polarization ellipticity perpendicular to direction of light propagation [139]. The authors used hexagonal-shaped particles and single cells trapped close to a gold-coated glass to generate a complete 360° pitch motion [139]. Shoji et al. [77] demonstrated 2D hexagonal arrays of particles created via induced plasmonic heating. The authors fabricated gold nanopyramidal dimmer arrays to transport particles via thermoplasmonic convection to the trapping sites [77].

### 4.3 Optothermophoretic effects for particle/biospecimen trapping

The key point to optically trap a single particle is to design a narrow and localised trapping potential well with a width comparable to the particle size. By using low concentration of suspended particles, the multiple particle trapping can be prevented, leading to an increase of the probability for single particle trapping. However, thermophobic particles (*S*_*T*_ > 0) can be easily repelled from the hotspot as the thermophoretic force diverges, indicating that efficient trapping of these particles is challenging. A smart optical design to overcome this obstacle was proposed by Braun et al. [127]. In this design, a laser beam was dynamically rotated along the outside of the metal structures to obtain a converged force field towards the cooler centre. Therefore, the high temperature gradient was localised near the boundary, while it remained low in the central region, leading to strong Brownian motion of the confined particle across the trapping region. A similar implementation was presented by Fränzl et al. [128], which allowed both the spatial confinement and observation of single amyloid fibril growth *in situ*, by varying the temperature of plasmonic nanostructures. An optical feedback scheme was developed to generate a steep temperature gradient capable of confining the motion of a single DNA molecule for several minutes [140]. The feedback was achieved by analysing the location of the biomolecule through CCD images while, simultaneously, the laser heating sites at the edge of the metal nanostructures were changed. This thermophoretic trapping method can provide a starting point for understanding various diseases related to protein misfolding.

Shoji et al. [141] combined the plasmon-enhanced optical force and thermophoresis to immobilise three different-sized DNA strands. Specifically, the DNA was optically separated and immobilised in double ring shapes, with the shorter and longer sized DNA forming the smaller and larger rings, respectively [141]. The authors mentioned that the mechanism creating these microrings was the Soret effect, caused by the large temperature gradient resulting from plasmonic excitation [141]. Nanoparticle sorting and trapping behaviour in two-dimensional optical lattices under the influence of optical and thermal effects have also been reported [142]. Under the influence of convection effects, nanoparticles captured by the plasmonic lattice are always attracted from the edges towards the central region of the lattice [142]. Note that the authors avoided using an adhesion layer to minimise the damping plasmon resonance and reduce the thermal contributions [142]. However, they reported a maximum transportation velocity of 5.7 μm/s for 500 nm and 8.5 μm/s for 100 nm particles, indicating the influence of these effects on the transport and trapping processes [142].

**Figure 6:**
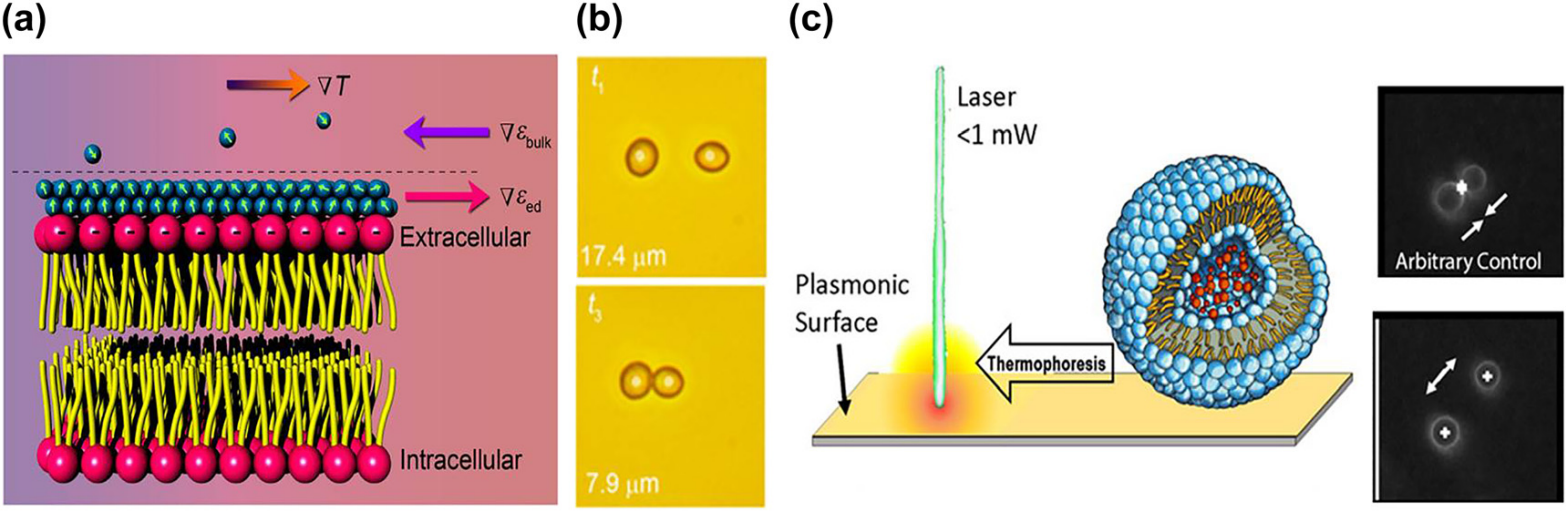
Optothermal trapping of biological particles. (a) Mechanism of cell trapping under a temperature gradient field ∇*T*, where ∇*ɛ*_ed_ is the permittivity gradient of the electric double layer and ∇*ɛ*_bulk_ is the permittivity gradient of the bulk water. (b) Precise manipulation of the distance between a pair of yeast cells captured at 100 nm resolution. Figures reproduced with permission from [130], Copyright 2017 ACS publications. (c) Left side image: a lipid vesicle trapped using plasmonic heating tweezers. Right side image: Video snapshots of dynamic manipulation of vesicles using a digital micromirror device to form arbitrary laser beam shapes. Figure reproduced with permission from [144], Copyright 2018 ACS publications.

The action of optothermophoretic tweezers has been demonstrated by illuminating a porous gold film substrate and creating a microscopic temperature gradient field to trap micro- or nanoparticles [101, 129]. These entropy-driven thermophoretic tweezers can also be used for the trapping and dynamic manipulation of biological cells [130, 143] and lipid vesicles [144] (Figure 6). Each phospholipid bilayer membrane is composed of a negatively charged hydrophilic group and two hydrophobic fatty acid tails (Figure 6(a)). The local electric field in the vicinity of the bilayer membrane results in an orientated layer of water molecules [130]. By manipulating the optothermal trapping potentials, biological cell or lipid vesicle orientation can be precisely controlled (Figure 6(b) and (c)).

### 4.4 Surfactant influence on particle trapping and motion

In most examples, at high temperature the stable trapping of the target particle is limited due to increased Brownian motion. Recently, Jiang et al. [75] studied the influence of the surfactant solution [41] on plasmonic optical trapping. They showed that the type of surfactant used to prevent particle agglomeration can affect the trapping performance by inducing a thermophilic or thermophobic response to the nanoparticles. For example, after the addition of sodium dodecyl sulfate (SDS), thermophoresis can assist the movement of nanoparticles towards the plasmonic hotspot [75]. On the other hand, Triton X-100 can produce a positive Soret coefficient that pushes the particles away from the trap potential well, confirming its negative role in the trapping process [75]. Additionally, for concentrations lower than the critical micelle concentration (CMC), when SDS was added to the colloidal particle solution the obtained trap stiffness was twenty times larger than that for non-ionic Triton X-100 [75]. In this work, the authors noted that, by optimising the surfactant conditions, the trapping process could be controlled for different plasmonic optical tweezers systems. Lin et al. [117] demonstrated metallic particle trapping by adding CTAC surfactant solution at concentrations slightly higher than the CMC. In their demonstration, all particles were absorbed by *CTA*^+^ cations and resulted in positively charged surfaces. After the gold nanostructured substrate was illuminated, local heating triggered both the ionic redistribution of *CTA*^+^ micelles and *Cl*^−^ anions [117]. Because the Soret coefficient of the micelles is higher than that of negative ions, the positively charged particles were directed and trapped at the plasmonic hotspot using low laser powers [117]. This work demonstrated the dynamic role of the surfactant in the trapping process when using micellar solutions and the applications of the thermo-electric forces. The absorbed surfactant molecules on the particle surface and substrate may result in an unconventional change in the electrical permittivity of the solvent between the particle and the metallic substrate [117].

Asymmetric, gold nano-antenna structures on glass substrates have been used to manipulate nanoparticles [102] suspended in cationic surfactant by optically controlling the subwavelength thermal hotspots. Specifically, a 300 nm polystyrene particle was transported on a triangle-shaped pattern of three gold nano-antennas by rotating the polarisation of the laser beam in an anticlockwise manner [102]. In this work, a femtosecond laser was used to modify the heat transfer and temperature profile between the nanoheater and the surrounding medium [145, 146]. Hence, femtosecond optical trapping has been demonstrated to effectively trap 500 nm polystyrene particles, revealing an enhancement in the trapping stiffness by a factor of five [102]. Moreover, by controlling the heat transfer using the ultrafast laser, the authors achieved precise manipulation of single CdSe/CdS core–shell quantum dots at the nano-antenna with tunable plasmon–exciton interactions [102].

To enable three-dimensional manipulation, photothermophoretic tweezers can also be developed using optical fibres [147, 148]. Kotnala et al. [147, 148] utilised optical fibres to demonstrate the optothermoelectric manipulation of 200 nm fluorescent polystyrene particles suspended in the cationic surfactant, CTAC [147]. For this purpose, they used a single-mode optical fibre with a thermoplasmonic tip, which converted the photons to phonons, leading to the formation of an optically controlled temperature gradient field [147]. By replacing the single-mode fibre with a multimode fibre, the authors achieved dynamic manipulation of 500 nm polystyrene particles [148]. The particle was trapped due to the thermoelectric force that arose from the cationic surfactant that was added to the solution [125]. In their implementation, the output of the optical fibre led to a speckle light pattern on a gold nano-island substrate that generated multiple thermal hotspots capable of simultaneously trapping more than one nanoparticle using low-power and unfocussed light [148]. In this trapping scheme, the speckle field intensities can control the drag force magnitude. The variation of light intensity influences the trapping behavior of the particles which depends on both the drag force and the thermoelectric force [148].

### 4.5 Particle polarisability can affect its motion

The polarisability of the particles is another parameter that may significantly affect optothermal manipulation and has not been explored fully. For example, in electrophoresis, the induced polarisability of colloidal particles depends on the magnitude of the electric current [149]. By controlling the magnitude of the current, it is possible to change the particle’s migration direction [149]. The results of this study [149] led to new directions of controlling particle manipulation based on the incident laser power that determines the amplitude of the optically generated optothermal field.

### 4.6 Opto-thermo-electrodynamic tweezers for particle and biomolecule manipulation

Trapping or sensing nanoparticles using single-unit plasmonic nanostructures, such as a double nanoholes, suffers from the issue of low-throughput because the particle-delivery process is frequently diffusion-limited [22]. Therefore, nanoparticles close to the plasmonic nanostructures can be effectively trapped [150]. Ndukaife et al. [72] developed opto-thermo-electrodynamic tweezers with the ability to rapidly transport nanometre-sized particles across large distances by combining an external electric field with optical and photo-induced heating forces. Specifically, the mechanism for generating this controllable load is based on the local gradient in the electric properties of the fluid induced by localised heating through the illuminated plasmonic nanoantenna [72]. Upon application of an external AC electric field in the presence of these gradients, an electrical force creates an impact on the drag force on the suspended particles and transports them to the plasmonic hotspot at significant speed. A similar technique has been recently demonstrated to trap biomolecules at femtomolar concentrations several micrometres away from the laser focus [151]. In this approach, an array of plasmonic nanoholes was illuminated and, simultaneously, a perpendicular AC electric field across the fluid was applied [151]. The plasmonic nanohole array resulted in the distortion of the applied field and generated two electric field components leading to two opposing microfluidic flows. Therefore, a stagnation zone was created far from the laser focus where biomolecules were effectively trapped for a long time [151].

### 4.7 Marangoni effect assists the trapping process

**Figure 7:**
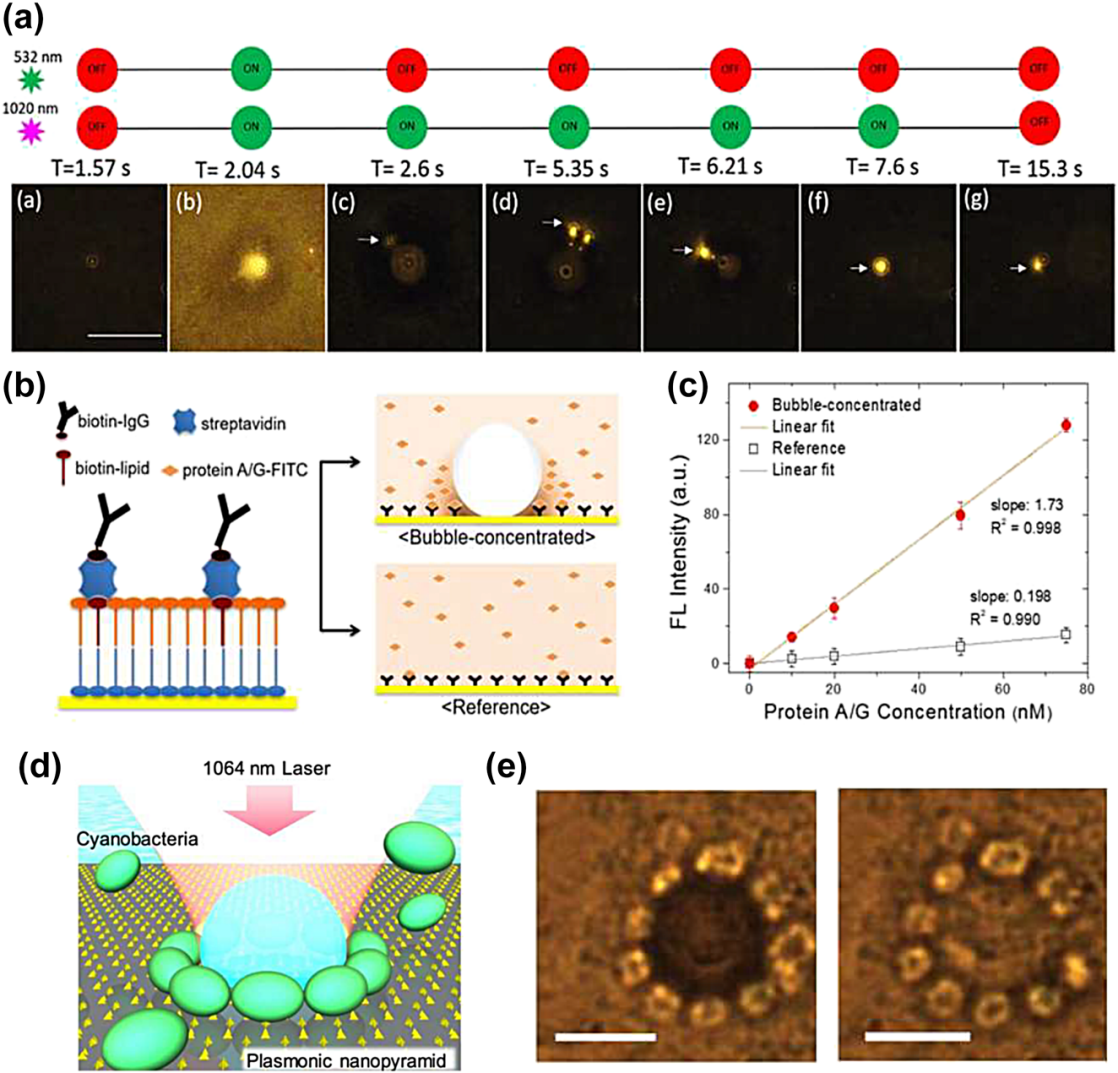
Marangoni-assisted trapping effect. (a) Video snapshot images of bubble-assisted trapping of 200 nm particles by illuminating a single nano-aperture with two laser beams, one for bubble generation and the other for particle trapping. Scale bar: 10 μm. Figures reproduced with permission from [125], Copyright 2019 ACS publications. (b) Left side image: IgG protein confined with biotin-streptavidin conjugation. Right side images: the protein localised at the bubble–solvent interface: diffusion-limited static incubation is presented as a reference. (c) The enhanced fluorescence intensity (FL) of the bubble system compared to the reference. Figures reproduced with permission from [152], Copyright 2020 ACS publications. (d) Schematic illustration of cyanobacteria trapping on a gold nanopyramid array with near infrared illumination, (e) Left side image: Optical trapping and fixing of bacteria caused by microbubble formation during illumination conditions. Right side image: after termination of the laser beam irradiation. The white line indicates 10 μm scale bar. Figures reproduced with permission from [153], Copyright 2020 ACS publications.

Optothermal manipulation based on microbubbles and plasmonic nanotweezers presents an advantage in precise control of the trapping process [125] and offers new functionalities, including opto-induced thermal assembly and printing [57, 154]. Nowadays, optothermal bubbles are widely used for rapid accumulation of dielectric particles [63], quantum dots [155], DNA [126] and bacteria [153, 156] leading to improved optical printing, sensing and chemical applications. Plasmonic heating of nano-apertures generated Marangoni convection flow, due to the surface tension gradient at the vapor–liquid interface. This type of flow has captured 200 nm particles at the bubble interface [125] (Figure 7(a)). The authors used two laser beams to control bubble generation and to trap dynamic colloidal nanoparticles [125]. This bubble-assisted trap significantly reduced the average particle trapping time from 30 min to several seconds [125]. In addition, microbubbles can be further exploited to assemble colloidal particles on plasmonic substrates, where they are immobilised because of van der Waals interactions [57]. Marangoni forces created by surface plasmon excitation using a Kretschmann configuration have also been used for opto-microfluidic manipulation of silicone oil and glycerol droplets [157]. The authors showed that the shape of the droplet was disturbed due to geometric asymmetries in the thermal gradient that extended beyond the region of plasmon coupling [157].

**Figure 8:**
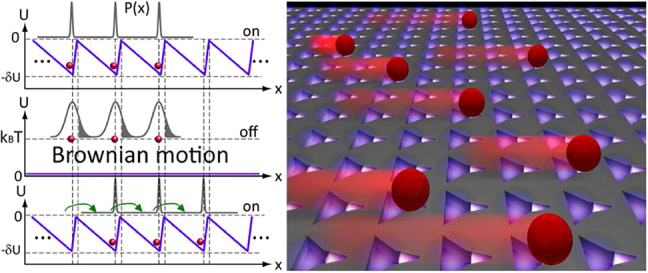
Brownian Ratchet. Left side image: Brownian motion can be rectified in the forward direction by the on–off modulation of a spatially asymmetric external potential. Blue and grey lines indicate potential and probability distribution, respectively. Right side image: schematic illustration of the Brownian ratchet principle where the motion of particles is determined by the asymmetric, modified triangular holes. Figures reproduced with permission from [160], Copyright 2016 ACS publications.

Recently, the manipulation of biomolecules based on the interfacial Marangoni effect has been reported [152, 158], see Figure 7(b) and (c). Using volatile substances with a water-immiscible liquid phase in a water environment is an efficient way to reduce the laser power required for bubble generation, hence enabling bulk-to substrate accumulation of proteins with minimal thermal deterioration of their activity [152]. Specifically, perfluorocarbons (PFCs) have been used as a volatile substance to form microbubbles at lower laser power [152] (Figure 7(b)). By modifying the plasmonic substrate with zwitterionic molecules, the capture efficiency of proteins and the diffusion limiting time have been improved [152]. An accumulation-assisted plasmonic chiral sensing system based on the Marangonic effect has been demonstrated for the label-free chirality detection of diabetes-related metabolic biomolecules [158]. For this purpose, plasmonic moiré chiral metamaterials [159] were fabricated to enable microbubble-induced accumulation of glucose and lactate metabolic biomolecules at concentrations less than 100 pM [158]. The authors mentioned that this approach could be used for point-of-care device for non-invasive screening and prognosis of early stage prediabetes or diabetes and its complications [158]. For microbiology applications, live cyanobacteria were trapped on an array of nanopyramids not by using the enhanced optical gradient force but rather through a mechanism similar to bubble lithography [153, 154] (Figure 7(d) and (e)). Microbubble formation enabled the transportation of bacteria to the interface between the bubble surface and the nanostructured substrate, where they were trapped without any alteration [153].

### 4.8 Brownian ratchet enables particle transportation

By harnessing thermal energy for directed motion, the implementation of Brownian ratchets [161–163] in engineered systems opens up potential applications in transporting or sorting nanoparticles [160] and biomolecules [164]. The principle of the Brownian ratchet refers to the effect where non-equilibrium fluctuations in an isothermal medium and an anisotropic system induce mechanical force and motion [161].

The plasmonic Brownian ratchet has been proposed [163] and demonstrated experimentally [160] by employing an array of plasmonic nanostructures with broken spatial symmetry to create a set of asymmetric potentials suitable for particle transportation. Initially, the particles were trapped in the potential minima (Figure 8). When the potential was turned off, the particles started to diffuse freely owing to thermal currents. When the trapping potential was turned on again, the particles were trapped by the neighboring trap site, resulting in net motion. Therefore, breaking the spatiotemporal symmetry of the system and combining the optical and thermal effects can result in precise control of particle motion in a fluid [160].

### 4.9 Dynamic control of the temperature profile

Ciraulo et al. [55] demonstrated an innovate platform to investigate the thermal landscape *in situ*. By combining digital holography microscopy with thermoplasmonic substrates, the authors clarified the various contributions from thermophoresis, thermo-osmosis, convection and radiation pressure in both the particle motion and the fluid dynamics [55]. Moreover, they noted that localised thermal perturbations at the microscale can lead to millimetre scale changes, which allow suspended particles to be transported over a long range [55]. The authors claimed that this approach will open new frontiers in fluid technology [55]. Durdevic et al. [165] demonstrated an optical technique to generate microscale temperature patterns with arbitrary shapes. The authors illuminated a uniform gold nanoparticle distribution using spatially contrasted laser beams to generate a desired temperature distribution [165].

## 5 Conclusions and future challenges

Optical manipulation has found numerous applications ranging from colloidal assembly to biological diagnosis. Although optical tweezers is the leading technique in this field, the combination of thermal and optical forces can has its own advantages. In the literature, several studies have been performed to elucidate the fundamental mechanisms of optothermal field formation and their influence on particle motion under temperature gradients [57], [58], [59, 166]. Recent research on plasmonic-assisted effects has provided further theories on the interplay between convection effects, optical forces, thermophoresis and particle motion which underpin newly developed thermoplasmonic technologies and applications [53]. Such plasmonic thermal effects offer tremendous possibilities in biomedical and clinical applications, enabling effective control of nanoscale thermal distributions and dissipation [53, 58]. Optothermal manipulation-assisted plasmonics can directly detect biomolecules [19, 151] and molecular interactions at the single-particle level and can be exploited for precise biodiagnostic processes. The local control of heating has also shown promise in medical applications like photothermal cancer therapy with reduced side effects [167]. However, the selection of the right type of particle for plasmonic nanoparticle based therapy is still challenging [166]. In one specific implementation, a gold nanostar particle was coated with a mesoporous silica shell and capped with a paraffin coat [168]. The mesoporous silica shell was loaded with doxorubicin. On irradiation, the gold nanostar can heat sufficiently for the paraffin coating to partially melt, thereby releasing the chemotherapeutic drug [168]. This type of particle was shown to efficiently compromise the viability of human breast cancer cells [168]. Maier et al. [169] demonstrated light-controlled guiding and injection of Janus nanopens into living cells, paving way to use such nanopens as nanocarriers for the spatially controlled injection of genetic materials into cells for gene therapy.

Despite the tremendous progress, additional research on the optothermophoretic mechanism is needed to clarify the role of and interaction phenomena among various solvents with different types of chemical bonding and ionic species with particle motion. For instance, it was reported that the motion of macro-ions was independent of the motion of mobile ions (known as counter ions) under a temperature gradient; however, this motion is not fully understood. In addition, DNA-coated polystyrene particles may respond differently to temperature gradients in the presence of surfactants. Surfactants can change the solvent particle interaction and the zeta potential of the particles which can cause DNA compaction on the particle surface [170]. DNA compaction can also affect the particle solvent and particle substrate interactions [170]. In another example, plasmonic substrates have shown unique advantages in the area of molecular diagnosis such as an ultrafast PCR test [158]. Such substrates are promising for *in vivo* biomedical applications, including lab-on-chip devices for early disease diagnostics.

One of the benefits of using optothermal manipulation is the requirement of low laser power owing to the dominance of thermophoresis over optical forces in this regime. However, when the laser power increases, the optical forces dominate simultaneously with several thermofluidic effects, indicating that the trap performance cannot be improved by simply increasing the optical power. In addition, convection effects appear at high temperatures where the thermophoretic effects dominate. Therefore, it is necessary to identify all the relevant parameters among several types of particle and plasmonic nanostructures and explore their interrelationships that are relevant for optothermal effects.

In this review, the optothermal effects generated by illuminating plasmonic nanostructures were presented. These phenomena offer a significant tool for engaging the temperature gradient with optical forces for dynamic particle manipulation systems. By designing on-demand plasmonic nanostructures, efficient and ultrafast thermodynamic motion on the nanoscale can be achieved. This review also included examples of applications in life science. Through coordinated management of laser power, heat and fluids via optothermo-mechanical coupling several unique techniques and applications in nanoscience can be developed in the future.
